# Supplementary material for: Increasing certainty in systems biology models using Bayesian multimodel inference
Source: Nat Commun. 2025 Aug 11;16:7416. doi: 10.1038/s41467-025-62415-4 (PMC12339951; doi:10.1038/s41467-025-62415-4)
Supplement: Supplementary file 1 — Supplementary Information [file 41467_2025_62415_MOESM1_ESM.pdf]

Supplementary Information: Increasing certainty in systems biology models using Bayesian multimodel inference

Nathaniel Linden-Santangeli,[1] Jin Zhang,[2] Boris Kramer,[1*] Padmini Rangamani[1,2*]

[1]Department of Mechanical and Aerospace Engineering, University of California San Diego,
9500 Gilman Dr, La Jolla, CA 92093, USA
[2]Department of Pharmacology, University of California San Diego,
9500 Gilman Dr, La Jolla, CA 92093, USA

[*]To whom correspondence should be addressed; E-mail: bmkramer@ucsd.edu;
prangamani@ucsd.edu.

Supplementary Figures

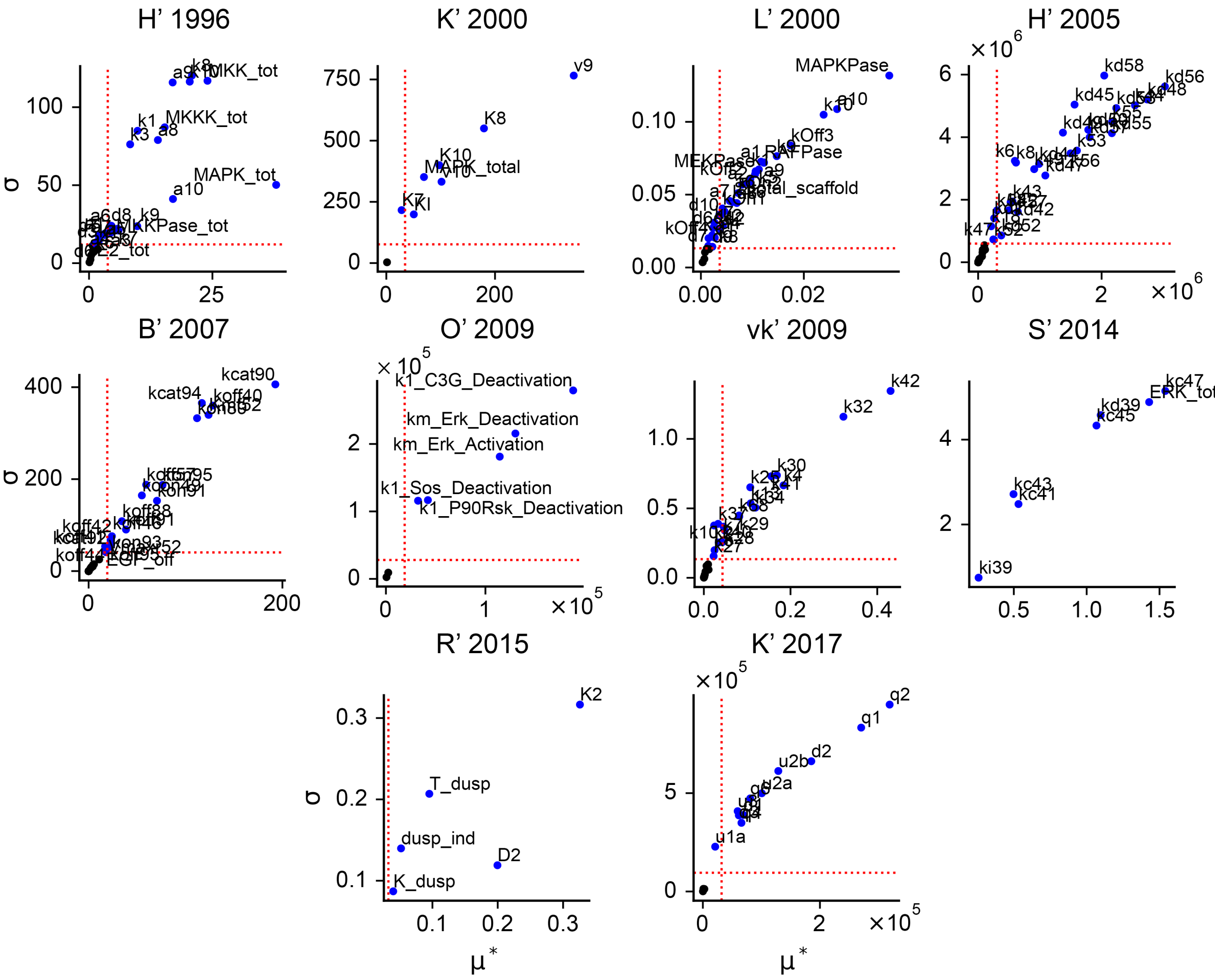

Supplementary Figure 1: **Morris sensitivity analysis reveals the parameters of ten ERK signaling models that most strongly influence ERK activity.** Scatter plots of Morris effect σ and μ^* normalized to the maximum of each effect independently for all models. Blue points with labels show *influential* parameters determined by $\sigma_i \leq 0.1$ or $\mu_i^* \geq 0.1$ for each parameter θ_i. Black points are *noninfluential* parameters that do not meet the thresholds.

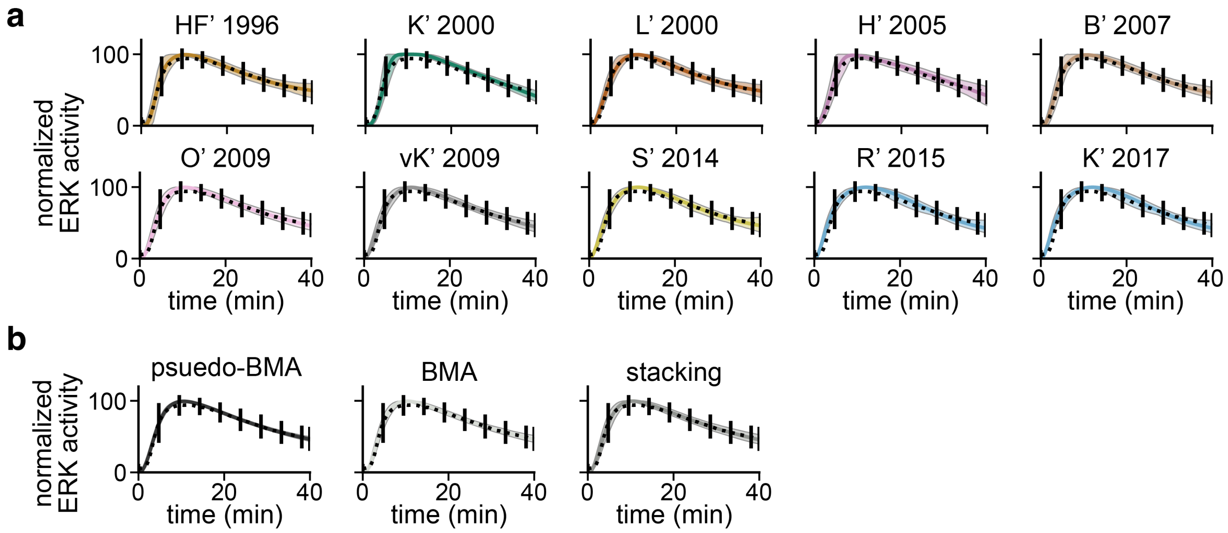

Supplementary Figure 2: **Posterior densities cytoplasmic ERK activity trajectory predictions.** (**a**) Posterior densities of cytoplasmic ERK activity trajectories for all models. (**b**)–(**d**) MMI predictions of cytoplasmic ERK activity using pseudo-BMA, stacking, and BMA, respectively.

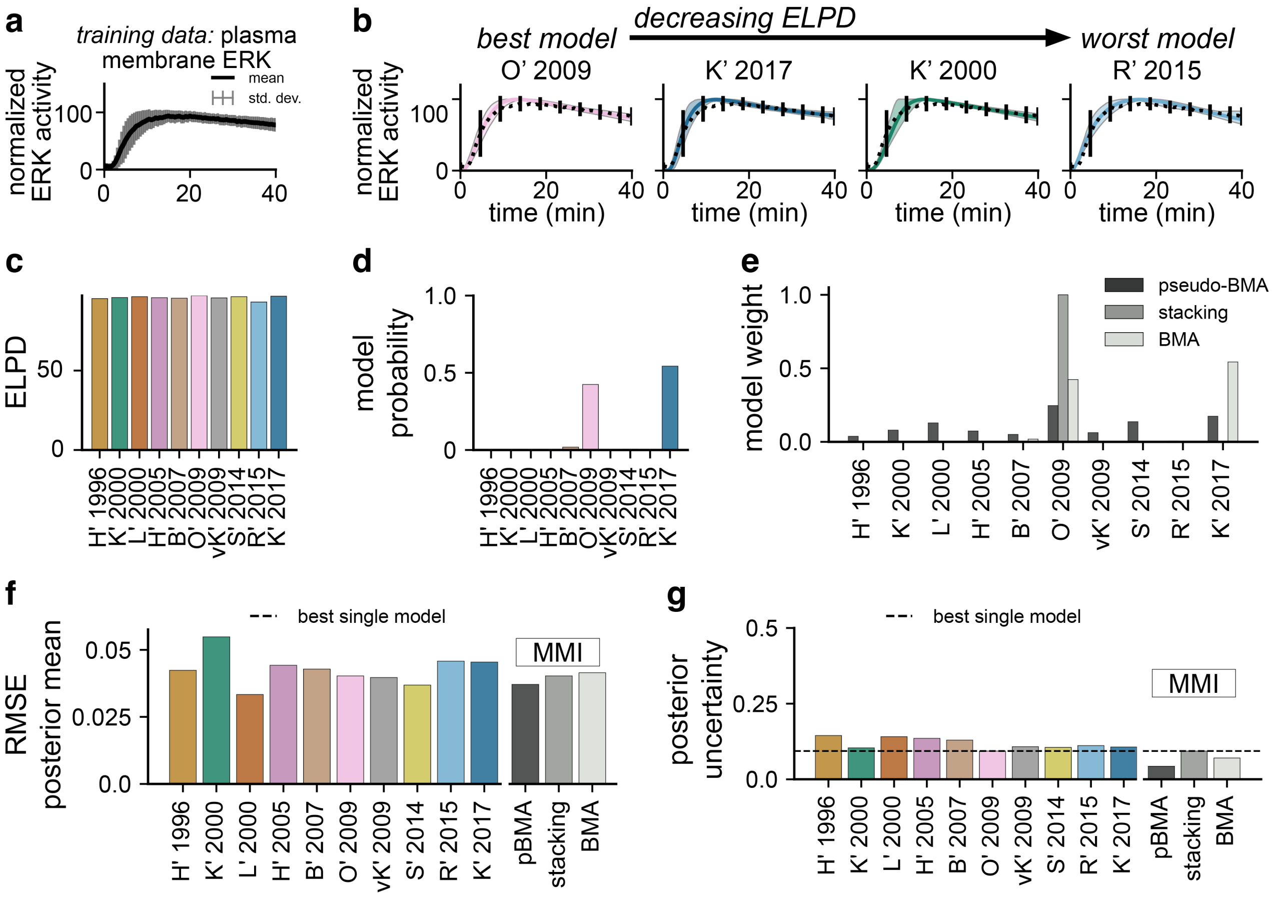

Supplementary Figure 3: **Multimodel inference reduces uncertainty in plasma membrane ERK activity predictions.** (**a**) Normalized experimental measurements of plasma membrane ERK activity. Mean (black trace) and standard deviation (grey bars) show statistics computed across all single-cell trajectories. Data originally presented in figure 1 of[1]. (**b**) Posterior densities of plasma membrane ERK activity trajectories for four out of nine models were ordered by decreasing ELPD. (**c**) ELPD values for all models. (**d**) Model probabilities for all models. (**e**) MMI model weights for all models using pseudo-BMA, BMA, and stacking. (**f**) RMSE of the posterior mean plasma membrane ERK activity predictions for each model and the multimodel predictions. The dashed black line shows the lowest RMSE of any single model. (**g**) Posterior uncertainty measured by the mean 95% credible interval width in time. The dashed black line shows the lowest uncertainty of any single model.

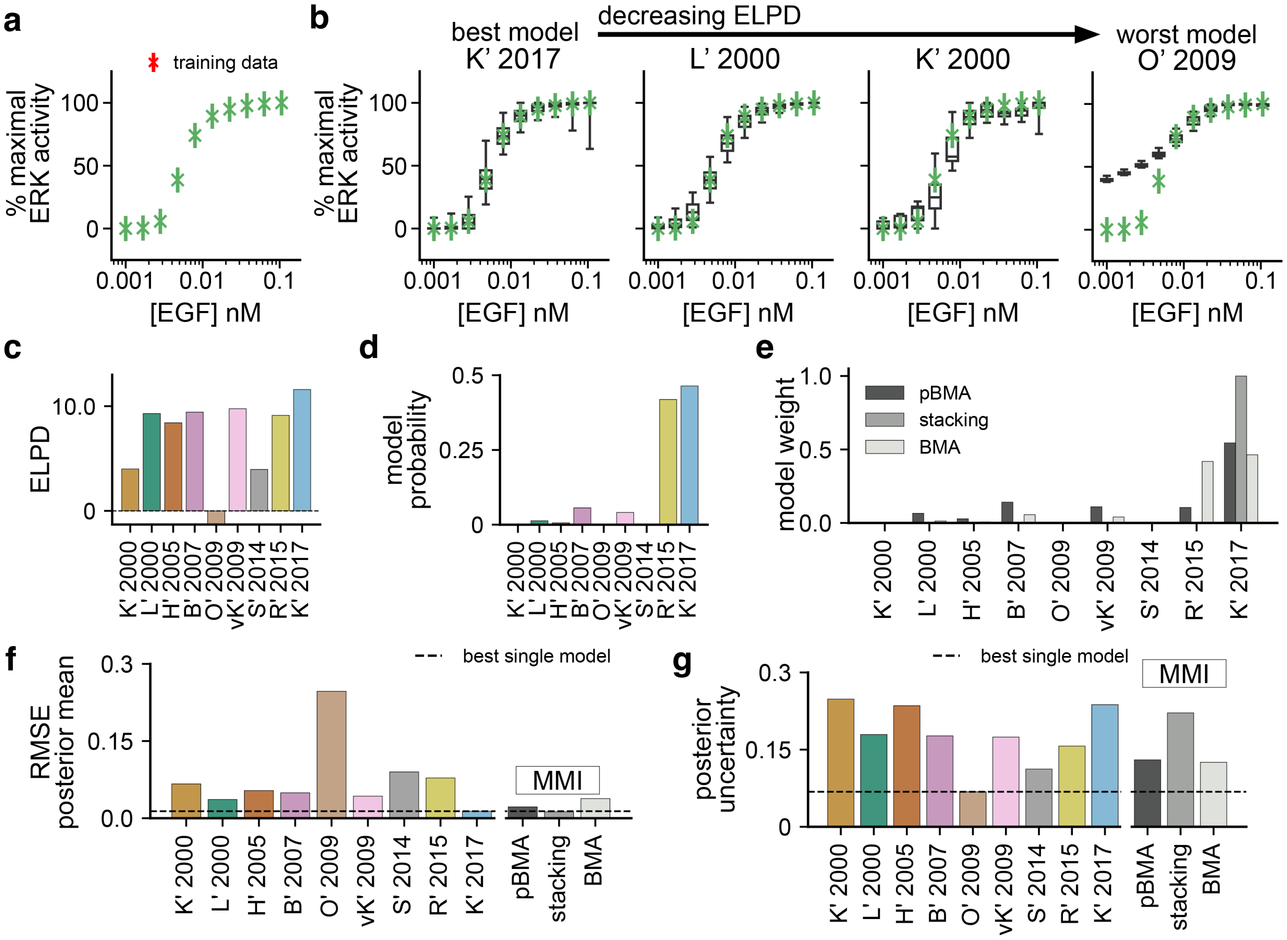

Supplementary Figure 4: **Multimodel inference of the EGF-ERK dose-response curve using synthetic training data.** (**a**) Synthetic EGF-ERK dose-response curve. Markers show the mean and error bars show plus or minus one standard deviation. (**b**) Posterior densities of the EGF-ERK dose-response for four out of nine models ordered by decreasing ELPD. Boxplots show the mean and interquartile range, and whiskers show the 95% credible interval. Data from A are reproduced in each panel. (**c**) ELPD values for all models. (**d**) Model probabilities for all models. (**e**) MMI model weights for all models using pseudo-BMA, BMA, and stacking. (**f**) RMSE of the posterior-predictive mean dose-response prediction for each model and the multimodel predictions. The dashed black line shows the lowest RMSE of any single model. (**g**) Posterior uncertainty is measured by the mean 95% credible interval width over all EGF levels. The dashed black line shows the lowest uncertainty of any single model.

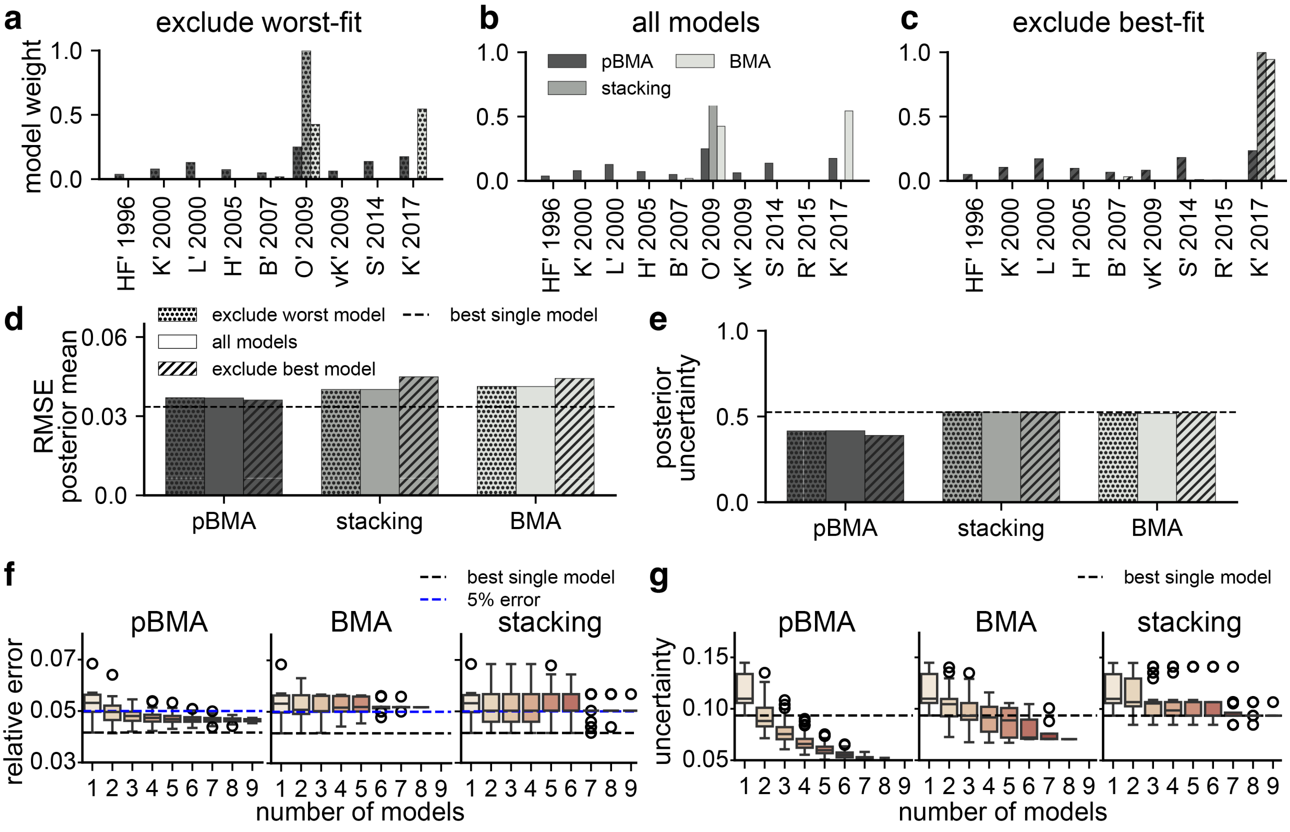

Supplementary Figure 5: **Bayesian multimodel inference is robust to perturbations in the set of plausible models for plasma membrane ERK activity trajectory predictions.** (**a**)–(**c**) Weights assigned to models in three model sets: (a) excluding the worst-fit model, R' 2015, (b) all models, (c) excluding the best-fit, O' 2009. (**d**) RMSE of the posterior mean cytoplasmic ERK activity prediction for each MMI method and model set. Dotted patterning corresponds to (a), no patterning to (b), and dashed patterning to (c). The dashed horizontal line is the RMSE of the model with the lowest average error, HF' 1996. (**e**) Posterior uncertainty is measured by the mean of the 95% credible interval taken over all EGF levels. (**f**) Relative error of the posterior mean for MMI predictions with increasing model set size. All possible combinations of models were tested at each size. The dashed blue line shows 5% relative error (0.05), and the dashed black line shows the lowest error of any single model, HF' 1996. (**g**) Average posterior uncertainty of ERK response for MMI predictions with increasing numbers of models. The dashed black line shows the uncertainty of the best model. (f),(g) Boxplots show the mean and interquartile range, and whiskers show the 95% credible interval. Open circles show outliers.

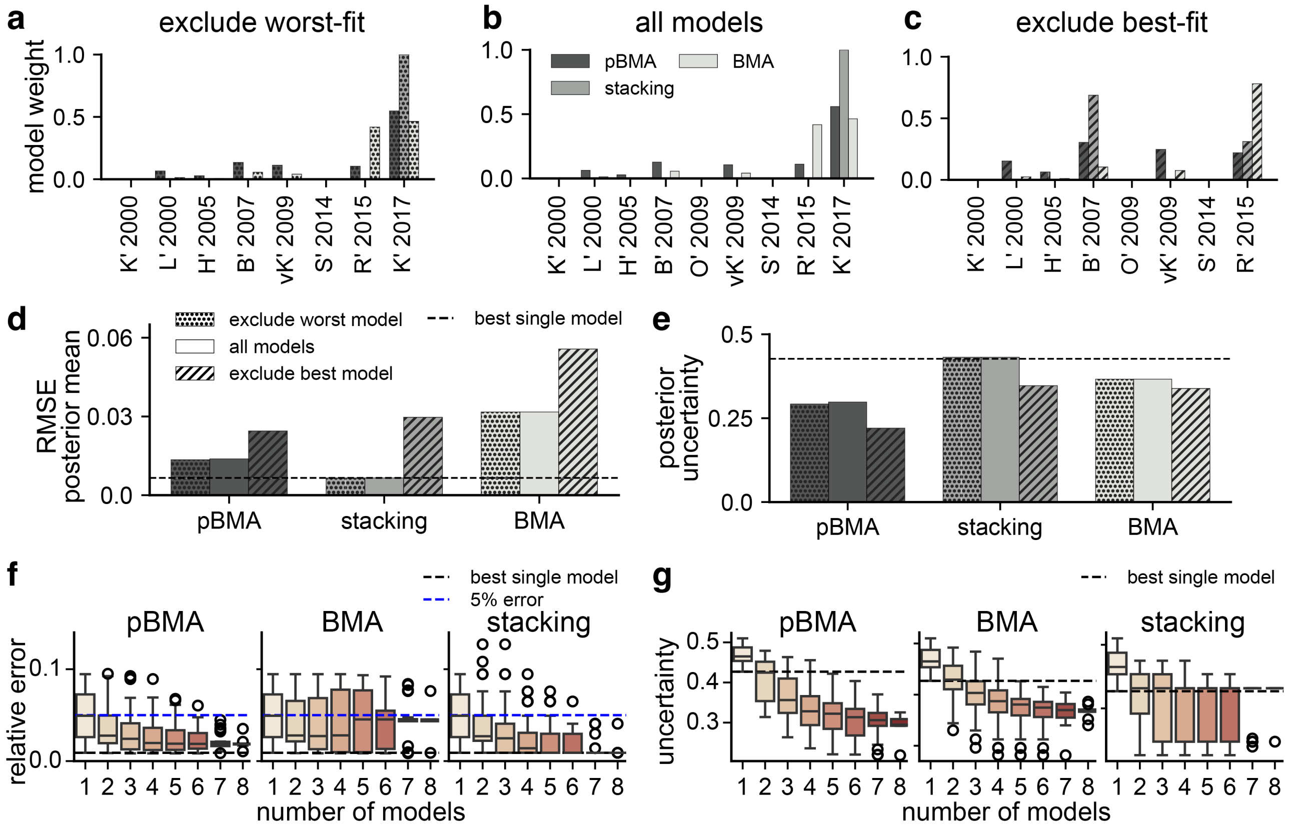

Supplementary Figure 6: **Bayesian multimodel inference is robust to perturbations in the set of plausible models for EGF-ERK dose-response predictions.** (**a**)–(**c**) Weights assigned to models in three model sets: (a) excluding the worst-fit model, O' 2009, (b) all models, (c) excluding the best-fit, K' 2017. (**d**) RMSE of the posterior mean dose-response prediction for each MMI method and model set. Dotted patterning corresponds to (a), no patterning to (b), and dashed patterning to (c). The dashed horizontal line is the RMSE of the best-fit model, K' 2017. (**e**) Posterior uncertainty is measured by the mean of the 95% credible interval taken over all EGF levels. (**f**) Relative error of the posterior mean for MMI predictions with increasing model set size. All possible combinations of models were tested at each size. The dashed blue line shows 5% relative error (0.05), and the dashed black line shows the lowest error of any single model, K' 2017. (**g**) Average posterior uncertainty of ERK response for MMI predictions with increasing numbers of models. The dashed black line shows the uncertainty of the best model, R' 2015. (f),(g) Boxplots show the mean and interquartile range, and whiskers show the 95% credible interval. Open circles show outliers.

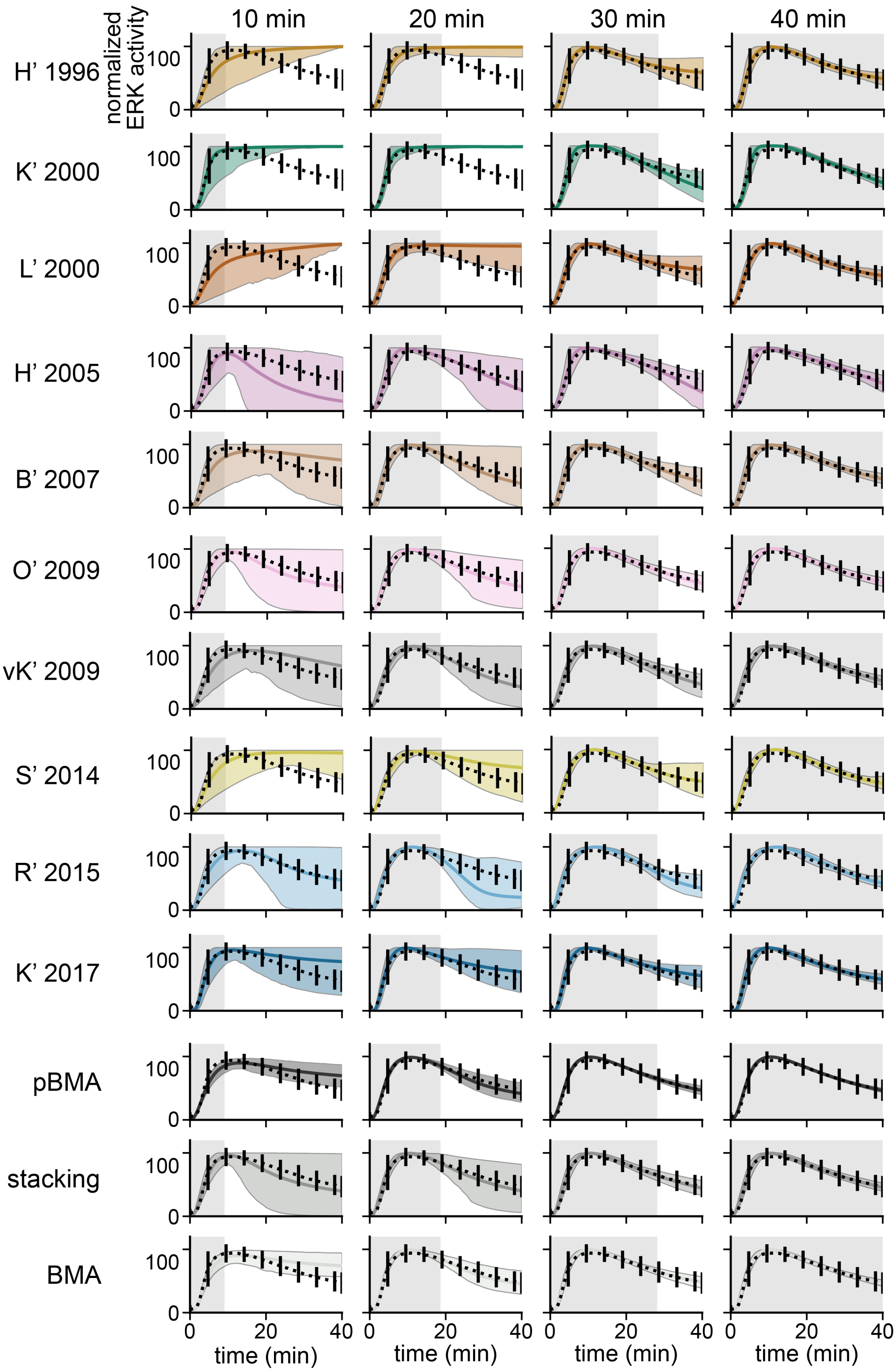

Supplementary Figure 7: **Posterior predictive densities of cytoplasmic ERK activity with shortened training data.** Black dashed line shows the data with error bars indicating the standard deviation. Solid colored line shows the posterior predictive mean trajectory. Shaded band shows the 95% credible interval of the posterior predictive density.

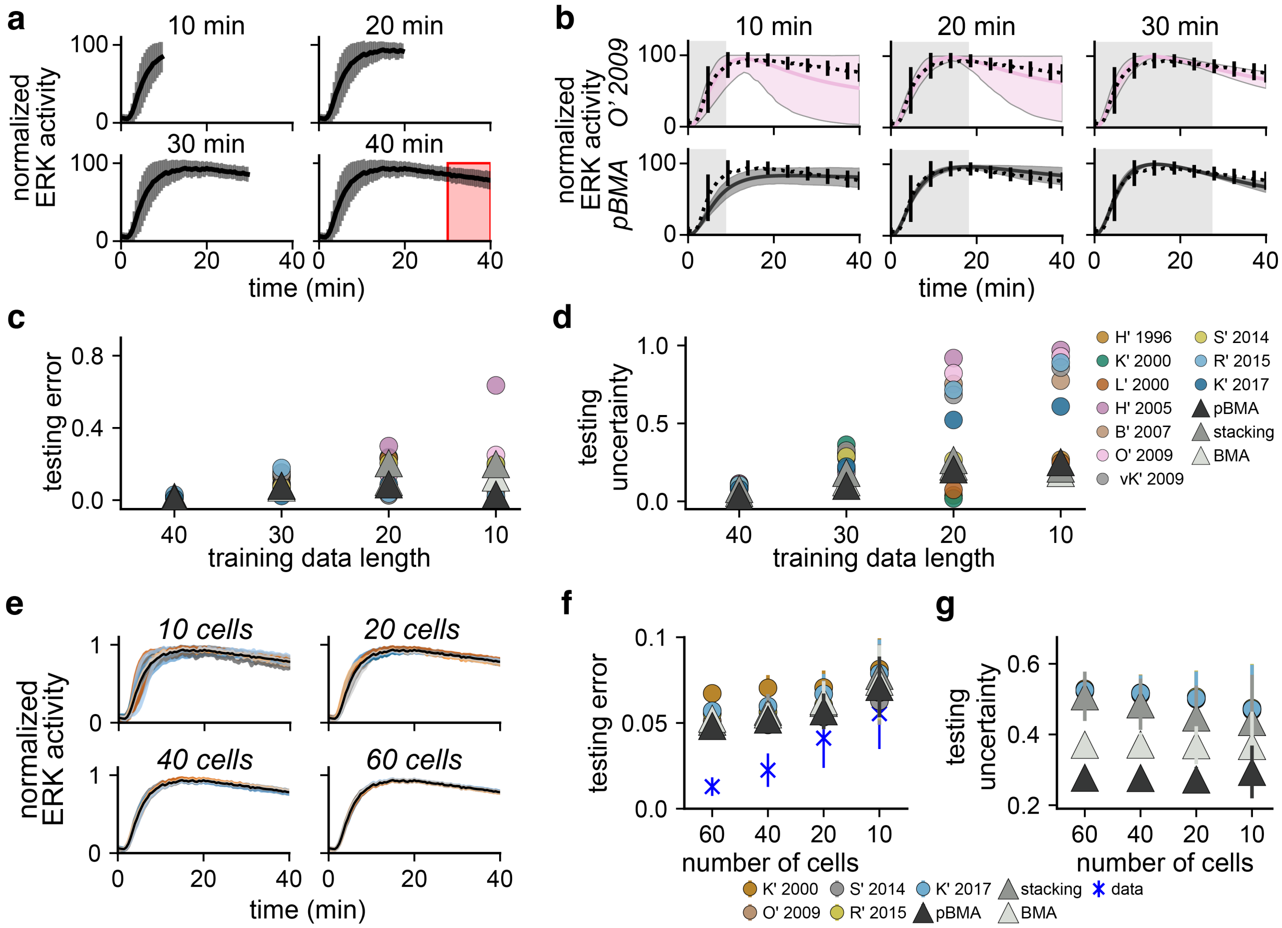

Supplementary Figure 8: **Mulitmodel inference predictions of plasma membrane ERK activity are robust to uncertainties due to decreasing data length and quality.** *Effects of decreasing data length.* (**a**) Shorter training data was constructed by truncating the original 40-minute plasma membrane ERK trajectories at the 10-, 20-, and 30-minute time points (mean, black trace; and standard deviation, grey bars). Predictive performance was assessed by computing errors and uncertainties in the final 10 minutes (red box). (**b**) Posterior predictions from decreased training data for the best model O' 2009 (highest ELPD across all training datasets) and MMI with pseudo-BMA. Predictions for additional models and MMI methods are shown in Supplementary Figure 7. (**c**) Predictive error (relative error) for the final 10 minutes ($t = 30 \to t = 40$ min) of plasma membrane ERK activity. (**d**) Predictive uncertainty (average width of the 95 % credible interval) for the final 10 minutes of plasma membrane ERK activity. (**e**) Lower-quality training data was generated by averaging over random subsets of 10, 20, 60, and 60 imaged cells. The black trace shows an original average of 76 cells. Colored traces show averages of 40 replicate random subsets. (**f**) The predictive error (relative error) of plasma membrane ERK activity with lower quality data compared to average activity trajectory using all cells. (**g**) Predictive uncertainty of plasma membrane ERK activity with lower quality data. (f),(g) Filled circles indicate average error of 40 replicates for individual models and triangles for MMI predictions. Error bars show the standard deviation over replicates. Blue markers show the error of the raw training data at each subset size compared to the original full-data mean.

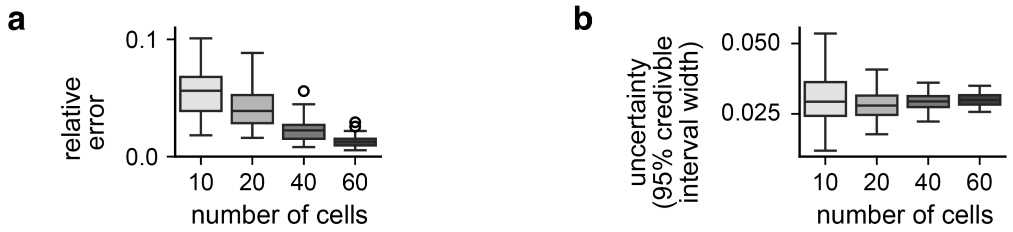

Supplementary Figure 9: **Error and uncertainty of lower-quality cytoplasmic data.** (**a**) Relative error of n=40 random subsets of 10, 20, 60, and 60 imaged cells compared to the original population average of 76 cells. (**b**) Average width of the 95% credible interval of n=40 random subsets of 10, 20, 60, and 60 imaged cells.

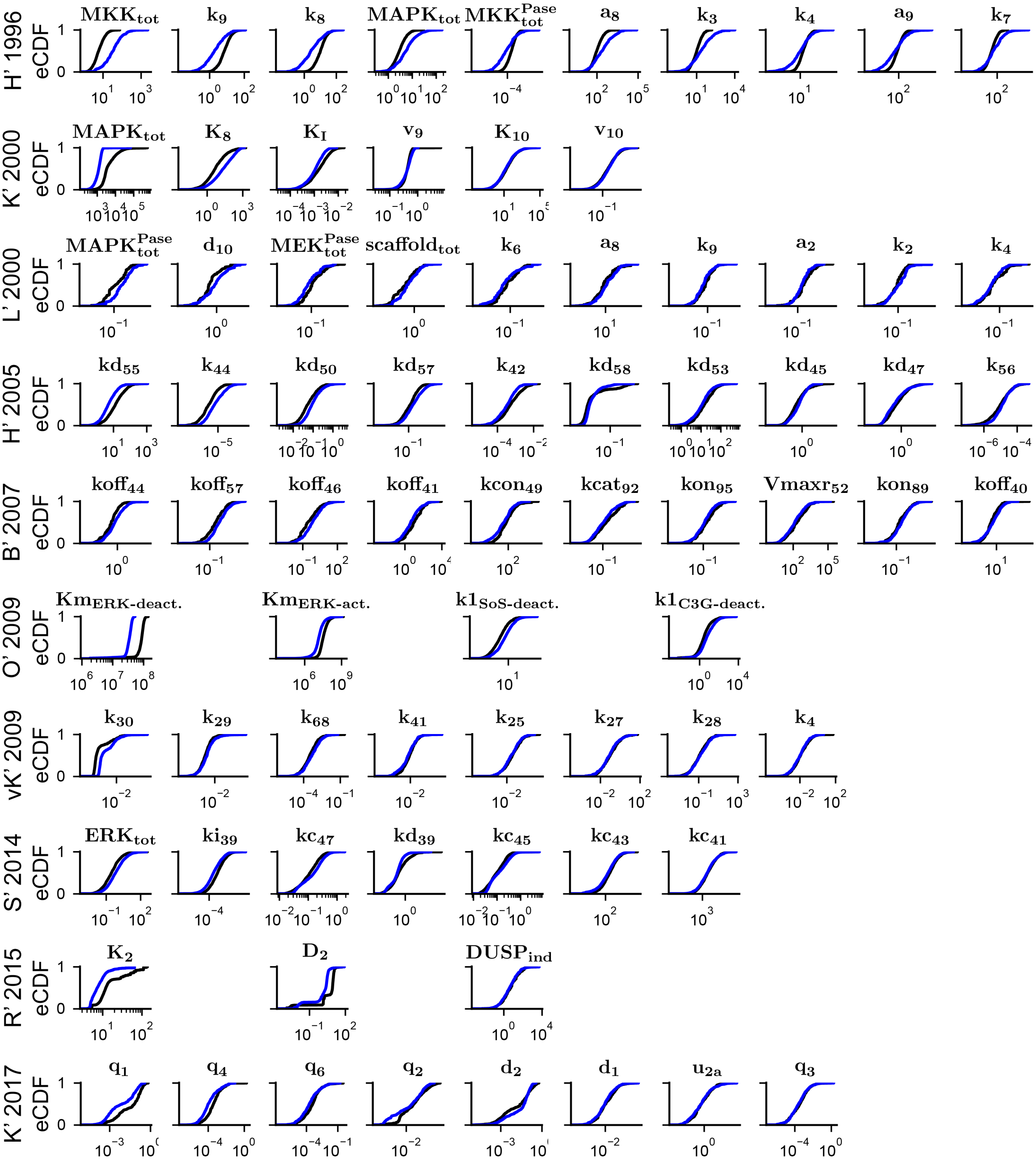

Supplementary Figure 10: **Differences in ERK model parameters can drive sub-cellular location-specific differences in ERK activity.** Figure shows estimated empirical cumulative density functions (eCDF) for the model parameters that showed the greatest variation between locations. All densities are statistically significantly different between locations ($p < 0.05$ by the Mann-Whitney U-test with a two-sided hypothesis). Blue indicates the cytoplasm, and black indicates the plasma membrane. Supplementary Table 3 lists the sample sizes for each model.

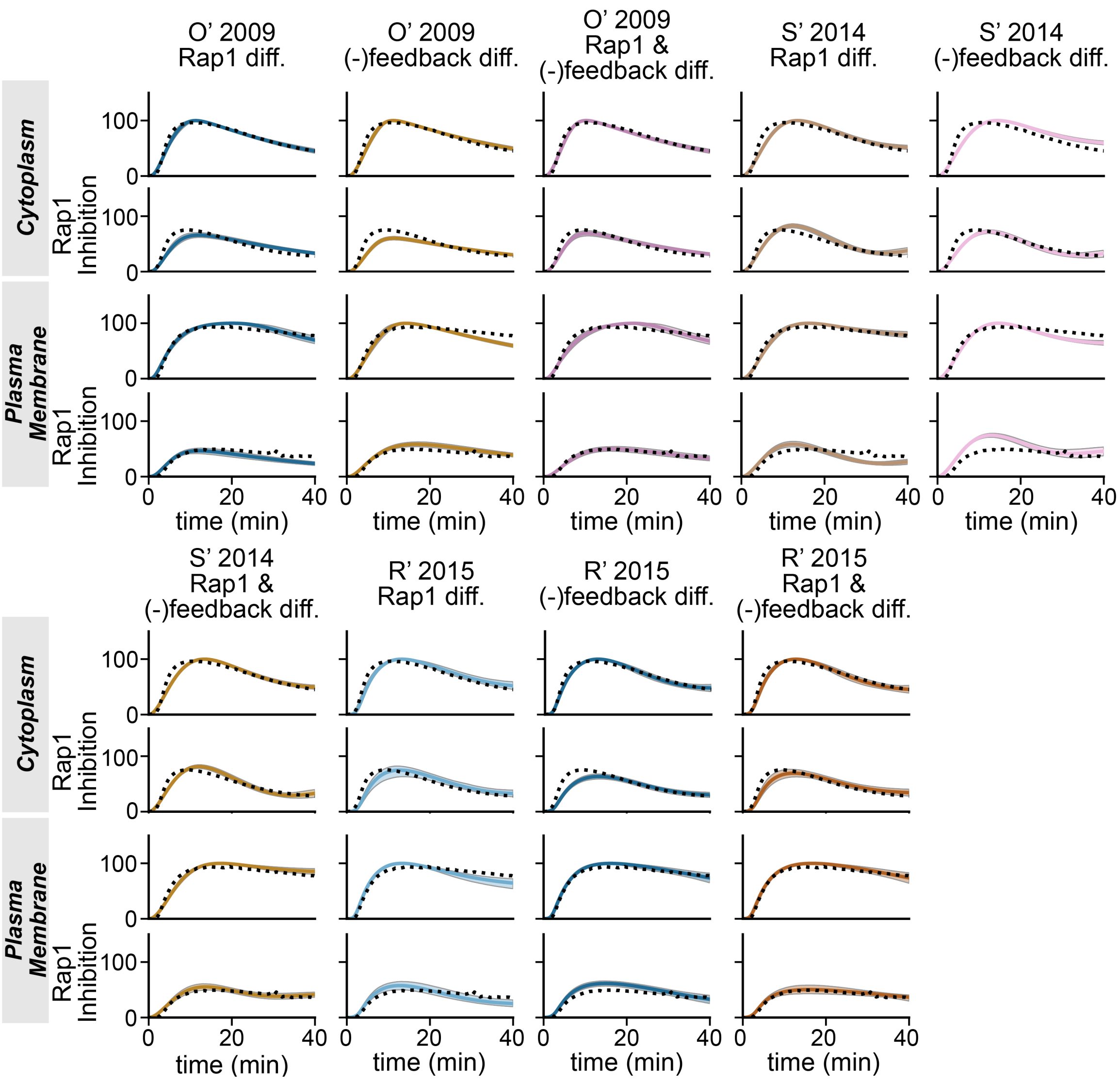

Supplementary Figure 11: **Posterior predictive densities of ERK activity under different location-specific hypotheses.** Black dashed line shows the data with error bars indicating the standard deviation. Solid colored line shows the posterior predictive mean trajectory. Shaded band shows the 95% credible interval of the posterior predictive density.

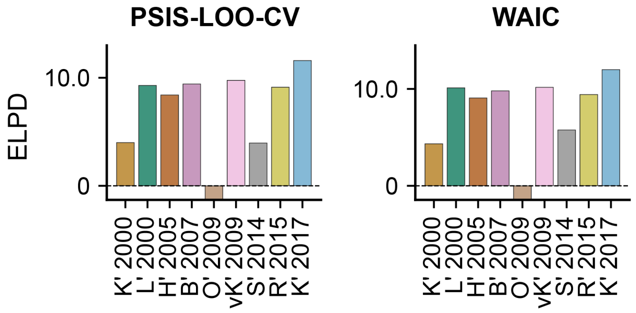

Supplementary Figure 12: **Comparison of ELPD predictions for synthetic dose-response data using PSIS-LOO-CV- and WAIC-based approximations.**

Supplementary Tables

Supplementary Table 1: **Set of extracellular signal-regulated kinase (ERK) signaling models used for Bayesian multimodel inference.** The number of state variables (# states (n_k)) represents the number of biochemical species in the model and the number of parameters (# parameters (p_k)) is the total number of free model parameters. The number of locally identifiable (# locally ID) is the number of parameters kept free after local identifiability analysis, and the number of sensitive parameters (# sensitive) is the number of parameters kept free after global sensitivity analysis.

Model Name	# states (n_k)	# parameters (p_k)	# locally ID	# sensitive
H' 1996[2]	16	36	36	26
K' 2000[3]	6	25	8	6
L' 2000[4]	27	40	40	25
H' 2005[5]	103	96	94	28
B' 2007[6]	117	216	90	20
O' 2009[7]	26	59	7	5
vK' 2009[8]	37	77	31	18
S' 2014[9]	7	20	7	7
R' 2015[10]	17	45	5	5
K' 2017[11]	35	23	14	11

Supplementary Table 2: **ODE solver and steady-state methods and corresponding hyperparameters for each ERK model.** We either use an ODE-solver or Newton iteration to find the steady-state solution. Steady-state ODE tolerances are used to assess the convergence of ODE-based solutions for either approach. We do not compute steady-states for the H' 1996 model, so we omit the corresponding hyperparameters. ODE-based solutions are solved until steady-state convergence or $t = t_{\text{max}}$.

Model	ODE tols (`atol`; `rtol`)	steady-state method	steady-state tols (`atol`; `rtol`)	time units	t_{max}
H' 1996	`1e-6`; `1e-6`	–	–	sec	–
K' 2000	`1e-6`; `1e-6`	Newton	`1e-6`; `1e-6`	sec	∞
L' 2000	`1e-6`; `1e-6`	ODE	`1e-10`; `1e-10`	sec	100,000 (s)
H' 2005	`1e-6`; `1e-6`	ODE	`1e-10`; `1e-10`	sec	12,000
B' 2007	`1e-6`; `1e-6`	ODE	`1e-5`; `1e-6`	sec	10,000
O' 2009	`1e-6`; `1e-6`	ODE	`1e-5`; `1e-6`	min	1,400
vK' 2009	`1e-6`; `1e-6`	ODE	`1e-5`; `1e-6`	sec	5,400
S' 2014	`1e-6`; `1e-6`	Newton	`1e-6`; `1e-6`	min	∞
R' 2015	`1e-6`; `1e-6`	Newton	`1e-5`; `1e-5`	min	540
K' 2017	`1e-6`; `1e-6`	Newton	`1e-6`; `1e-6`	sec	10,800

Supplementary Table 3: **Sampling runtimes and sample counts for all Sequential Monte Carlo sampling.** Wall-clock times are shown in hours. Parentheses show: (# of chains and # number of samples/chain) if those values differ from: # of chains = 4 and # number of samples/chain = 500, i.e. (4; 500). Sampling for Figure 3 and Supplementary Figures 3–4 was performed on a Linux Workstation with dual Intel Xeon 2.60GHz processors and 123Gb of DDR3 RAM. Sampling for Figure 6 was performed on a Macbook Pro with the Apple M3 Pro CPU and 18Gb of DDR5 RAM.

**Model**	Figure 3	Supp. Figure 3	Supp. Figure 4	Figure 6
H' 1996	**10.68**	**0.24**	-	-
K' 2000	**0.04**	**0.04**	**1.72** $(4; 1000)$	-
L' 2000	**0.07**	**0.08**	**8.65** $(4; 200)$	-
H' 2005	**1.97** $(8; 100)$	**1.88** $(8; 100)$	**110.66** $(4; 100)$	-
B' 2007	**1.73**	**1.77**	**167.87** $(4; 100)$	-
O' 2009	**0.11**	**0.12**	**2.10** $(4; 250)$	-
vK' 2009	**0.31**	**0.40**	**0.86** $(4; 100)$	-
S' 2014	**0.07**	**0.07**	**16.90** $(4; 1000)$	-
R' 2015	**0.04**	**0.04**	**18.64** $(4; 1000)$	-
K' 2017	**0.19**	**0.22**	**222.76** $(4; 1000)$	-
O' 2009 Rap1 diff.	-	-	-	**0.66**
O' 2009 (-)FB diff.	-	-	-	**0.58**
O' 2009 Rap1 and (-)FB diff.	-	-	-	**0.65**
S' 2014 Rap1 diff.	-	-	-	**0.21**
S' 2014 (-)FB diff.	-	-	-	**0.16**
S' 2014 Rap1 and (-)FB diff.	-	-	-	**0.19**
R' 2015 Rap1 diff.	-	-	-	**0.37**
R' 2015 (-)FB diff.	-	-	-	**0.33**
R' 2015 Rap1 and (-)FB diff.	-	-	-	**0.66**

Supplementary Table 4: **Model predictive performance (ELPD) and MMI weights of the top and bottom ten models of the 94 total compartment-specific parameter combinations.** Bold entries indicate weight greater than 0.01 by any MMI method.

Model	ELPD	pseudo-BMA weight	stacking weight	model probability
Top ten models:				
**O' 2009 Rap1, ppMEK-ERK & ppERK-pASE diff.**	**343.585**	**0.404**	**0.882**	**0.343**
**O' 2009 all diff.**	**343.406**	**0.337**	**0.0**	**0.093**
**O' 2009 Rap1, (-)FB & ppERK-pASE diff.**	**341.456**	**0.072**	**0.0**	**0.16**
**O' 2009 Rap1 & ppERK-pASE diff.**	**341.371**	**0.08**	**0.0**	**0.402**
**R' 2015 Rap1 & (-)FB diff.**	**339.737**	**0.065**	**0.054**	**0.0**
**R' 2015 Rap1, (-)FB & ppERK-pASE diff.**	**337.448**	**0.037**	**0.063**	**0.0**
O' 2009 (-)FB, ppMEK-ERK & ppERK-pASE diff.	337.233	0.002	0.0	0.001
O' 2009 Rap1 & (-)FB diff.	336.723	0.001	0.0	0.001
O' 2009 Rap1, (-)FB & ppMEK-ERK diff.	335.841	0.0	0.0	0.0
S' 2014 Rap1, (-)FB, Raf-deact. & ppERK-dephos. diff.	335.068	0.0	0.0	0.0
Bottom ten models:				
R' 2015 all diff.	278.966	0.0	0.0	0.0
R' 2015 (-)FB & ppERK-pASE diff.	276.007	0.0	0.0	0.0
S' 2014 (-)FB & Raf-deact. diff.	262.615	0.0	0.0	0.0
S' 2014 Raf-deact. diff.	260.414	0.0	0.0	0.0
S' 2014 (-)FB diff.	245.325	0.0	0.0	0.0
R' 2015 ppMEK-ERK diff.	208.882	0.0	0.0	0.0
R' 2015 (-)FB & ppMEK-ERK diff.	201.588	0.0	0.0	0.0
R' 2015 (-)FB, ppMEK-ERK & ppERK-pASE diff.	201.08	0.0	0.0	0.0
R' 2015 Rap1, ppMEK-ERK & ppERK-pASE diff.	175.419	0.0	0.0	0.0
R' 2015 ppMEK-ERK & ppERK-pASE diff.	172.626	0.0	0.0	0.0

Supplementary Files

We provide an Excel file that details the nominal parameters and initial conditions for all 10 ERK signaling models used in this work. The file is available for download from our GitHub repository which is accessible here: `https://github.com/RangamaniLabUCSD/multimodel-inference/blob/main/results/model_info_supplemental_material.xlsx`.

References

1. Keyes, J. *et al.* Signaling diversity enabled by Rap1-regulated plasma membrane ERK with distinct temporal dynamics. *Elife* **9** (May 2020).
2. Huang, C. Y. & Ferrell Jr, J. E. Ultrasensitivity in the mitogen-activated protein kinase cascade. *Proc. Natl. Acad. Sci. U. S. A.* **93,** 10078–10083 (Sept. 1996).
3. Kholodenko, B. N. Negative feedback and ultrasensitivity can bring about oscillations in the mitogen-activated protein kinase cascades. *Eur. J. Biochem.* **267,** 1583–1588 (Mar. 2000).
4. Levchenko, A., Bruck, J. & Sternberg, P. W. Scaffold proteins may biphasically affect the levels of mitogen-activated protein kinase signaling and reduce its threshold properties. *Proc. Natl. Acad. Sci. U. S. A.* **97,** 5818–5823 (May 2000).
5. Hornberg, J. J. *et al.* Control of MAPK signalling: from complexity to what really matters. *Oncogene* **24,** 5533–5542 (Aug. 2005).
6. Birtwistle, M. R. *et al.* Ligand-dependent responses of the ErbB signaling network: experimental and modeling analyses. *Mol. Syst. Biol.* **3,** 144 (Nov. 2007).
7. Orton, R. J. *et al.* Computational modelling of cancerous mutations in the EGFR/ERK signalling pathway. *BMC Syst. Biol.* **3,** 100 (Oct. 2009).
8. Von Kriegsheim, A. *et al.* Cell fate decisions are specified by the dynamic ERK interactome. *Nat. Cell Biol.* **11,** 1458–1464 (Dec. 2009).
9. Shin, S.-Y. *et al.* The switching role of β-adrenergic receptor signalling in cell survival or death decision of cardiomyocytes. *Nat. Commun.* **5,** 5777 (Dec. 2014).
10. Ryu, H. *et al.* Frequency modulation of ERK activation dynamics rewires cell fate. *Mol. Syst. Biol.* **11,** 838 (Nov. 2015).
11. Kochańczyk, M. *et al.* Relaxation oscillations and hierarchy of feedbacks in MAPK signaling. *Sci. Rep.* **7,** 38244 (Jan. 2017).
